# The Past, Present and Future of Geodemographic Research in the United States and United Kingdom

**DOI:** 10.1080/00330124.2013.8487642345

**Published:** 2013-11-27

**Authors:** Alexander D. Singleton, Seth E. Spielman

**Affiliations:** University of Liverpool; University of Colorado at Boulder

**Keywords:** geodemographics, GIS, social area analysis, urban geography

## Abstract

This article presents an extensive comparative review of the emergence and application of geodemographics in both the United States and United Kingdom, situating them as an extension of earlier empirically driven models of urban socio-spatial structure. The empirical and theoretical basis for this generalization technique is also considered. Findings demonstrate critical differences in both the application and development of geodemographics between the United States and United Kingdom resulting from their diverging histories, variable data economies, and availability of academic or free classifications. Finally, current methodological research is reviewed, linking this discussion prospectively to the changing spatial data economy in both the United States and United Kingdom.

Geodemographic classifications organize areas into categories sharing similarities across multiple socioeconomic attributes. These classifications have either a national extent or localized focus (e.g., a region) and are built to describe the generalities of places or to examine the geography of specific domains of interest (e.g., health). Within a geodemographic typology, each cluster is identified from a distinctive collection of attributes; for example, wealthy neighborhoods, where most households comprise older individuals living within apartments. Clusters are typically named by the classification builder (e.g., Elderly Suburbs) and are accompanied by rich media descriptions. An example geodemographic classification for the United States is shown in Figure 1.

Geodemographic classifications tend to be highly dimensional, typically including anywhere from a dozen to several hundred empirically derived characteristics. Using many variables to construct a set of meaningful categories can be a complex process, and there is an extensive literature on both the selection of input variables and the analytical methods for creating classifications. The literature reflecting on the use of geodemographic systems, however, is less well developed. As such, in this article we take an international and comparative perspective, specifically considering the evolution of geodemographics in the United States and the United Kingdom. In spite of similar origins in the mid-1970s and early historical parallels, geodemographic classifications are used quite differently between the two countries. In this article we consider the historical evolution of geodemographic classifications in the United States and the United Kingdom, the current geodemographic market, and the near-term prospects for geodemographic classification given recent changes and pressures on national statistical systems.

## The Evolution of Geodemographics in the United States and United Kingdom

Geodemographic analysis has its origins in the work of human ecologists in the 1920s and 1930s, and its history includes the large body of work in social area analysis and factorial ecology (see Timms [1971] and Rees [1972] for extensive reviews). The representations created by factorial ecology and social area analysis attempted to reduce the complexities of human settlements into simplified typologies (Abler, Adams, and Gould 1971) and, as such, provide the conceptual and theoretical foundations for geodemographics. Social area analysis, as originally conceived by Shevky and Williams (1949), measured society at a census tract scale along three dimensions: urbanization, social rank, and segregation. Later, Shevky and Bell (1955) presented these measures within a more extensive theoretical framework. Their work, however, has been criticized (Robson 1969) as a post facto rationalization of Shevky and Williams (1949). Alongside other criticisms about the applicability of general frameworks within specific regional contexts, this led to the emergence of city-specific factor-analytic models, which Berry and Kasarda (1977) described as factoral ecologies. Social area analysis and factoral ecology both represent an early data-driven ideographic form of science, and their key contribution was to derive a set of methods that could be applied to illustrate urban socio-spatial structure.

Geodemographic classification emerged later (Tryon 1955; Webber 1975) as a methodological solution for handling highly dimensional census data (Webber 1978) and employed clustering techniques to deliver categorical descriptors of small-area geography. Early examples focused on single-city studies; however, these were later expanded into national coverage classifications (Webber and Craig 1976, 1978; Webber 1977).

By the mid-1970s, commercial interest in the potential of geodemographic analysis had emerged on both sides of the Atlantic. Within the United Kingdom, the national census ward–level classification produced by Webber (1977) began to be marketed by the U.S. company CACI under the brand name ACORN (A Classification of Residential Neighborhoods; Harris, Sleight, and Webber 2005). In parallel, in the United States, Robbins created the PRIZM (Potential Rating Index for ZIP Markets) classification in 1974, which linked census and lifestyle survey data to the then newly created ZIP codes (Weiss 1988). Throughout the 1980s, geodemographics gained traction as a private-sector marketing tool (Reibel 2011), and the number of commercial classifications being offered in the United Kingdom and the United States burgeoned. The details of this growth are adequately documented elsewhere (Beaumont and Inglis 1989; Flowerdew and Goldstein 1989; Batey and Brown 1995; Sleight 1997; Harris, Sleight, and Webber 2005).

Over time, both the U.S. and UK markets have expanded and diversified, with those national classifications existing as of 2012 summarized in Tables 1 and 2. The classifications listed are those ascribed to areas rather than smaller taxonomic units such as individuals or households and exclude classifications built for specific purposes (e.g., health). Furthermore, where data are missing from these tables, these details were either not in the public domain or were withheld by the commercial providers.

In both countries there is a diverse set of geodemographics, with the United Kingdom having a greater variety of classifications supplied by small-to medium-sized enterprises (SMEs). Classifications in the United States included a maximum of two hierarchical levels, whereas in the United Kingdom, a number of classifications included a third level. In both countries, however, a limited number of providers advertise additional microlevel versions of their classifications that enable the appending of client-specific data and reclustering of the taxonomic units into new bespoke segmentations. The addition of hierarchies provides greater flexibility in how classifications can be used. More aggregate groupings enable the profiling of data with restricted numbers of cases and can reduce the standard errors of some variables that are imprecise at finer levels of aggregation (bringing them within usable limits); however, the coarser groupings improve attribute resolution at the expense of geographic resolution and reduce the utility of a classification for identifying interesting patterns.

Differences in cluster frequency between commercial and noncommercial providers likely relate to the types of data available to commercial organizations (e.g., commercial survey data), thus enabling areas to be profiled over a greater number of dimensions and increasing the possibility that additional groupings of similarity might emerge from a cluster analysis. The units used to build the classifications in the United States were most prevalently census block group level (comprising between 600 and 3,000 people, with an optimum size of 1,500), which are much more aggregate than those typically used in the United Kingdom (e.g., 2001 output area, comprising between 100 and 4,000 people, with an optimal size of more than 125 households). A caveat is that the data used to build geodemographics can be drawn from a variety of scales; however, the units detailed in Tables 1 and 2 relate where possible to those units used as the building blocks of the classifications. When classifying larger geographic units, heterogeneity that might exist at finer scales can be smoothed away; on the other hand, finer scale data are often subject to significant sampling error. It is interesting to find that classifications within the United States typically have a greater average number of maximum clusters than in the United Kingdom. These differences are influenced by the data available or used and also those choices of the classification builders, which might be influenced by the norms of competitors within their respective national markets.

In the United Kingdom there has been a long history of free national classifications. This is a critical difference between the two countries and, as discussed in the next section, seems to have significantly affected uptake of geodemographic methods in the U.S. academic sector. Academics in the United Kingdom have published classifications corresponding to the decennial release of the 1981 (Charlton, Openshaw, and Wymer 1985), 1991 (Blake and Openshaw 1994, 1995), and 2001 (Vickers and Rees 2007) censuses. More recently within the United States, a free classification has been built using the 2000 Census (http://worldclusters.org/?pageid=22), but this does not appear within the peer-reviewed literature. A further U.S. geodemographic example (although not named as such) appeared in the context of health as an eight-cluster segmentation of counties for comparison of mortality disparities (Murray et al. 2006) and, at a coarser scale, work identifying regions in the United States (Garreau 1992).

Openshaw (1983) argued that because cluster analysis is an exploratory data analysis technique, “[a] classification can only be deemed ‘good’ or ‘poor’ when it has been evaluated in terms of the specific purpose for which it is required; there is no magic universal statistical test that can be applied nor is there any possibility of deriving a classification suitable for all purposes” (245). This criticism is problematic for national geodemographic classifications given the wide variety of potential applications and localities in which they can be applied. Openshaw, Cullingford, and Gillard (1980) made a comparison between a locally and nationally produced classification, finding that the representations created were quite different. This fact alone, though, does not necessarily undermine the validity or usefulness of national classifications. It simply means that the inputs of the two classifications are comparing attributes against different sets of denominators (Webber 1980) and for different purposes. A similar critique emerged in the United States, raising concerns over the applicability of utilizing the PRIZM classification (then from Claritas) with nationally collected survey data to target at local scales (Atlas 1989). Later work has argued that “differences between [geodemographic] classes are generally smaller than the differences found within any particular class” (Voas and Williamson 2001, 74), calling again for geodemographics to be task specific, thus echoing the earlier concerns of Openshaw, Cullingford, and Gillard (1980) and Openshaw (1983). The ability to construct bespoke classification has, however, improved significantly in recent years as spatial data infrastructures have matured (Singleton and Longley 2009; Adnan et al. 2010).

## Academic Applications of Geodemographics in the United States and United Kingdom

**Figure 2:**
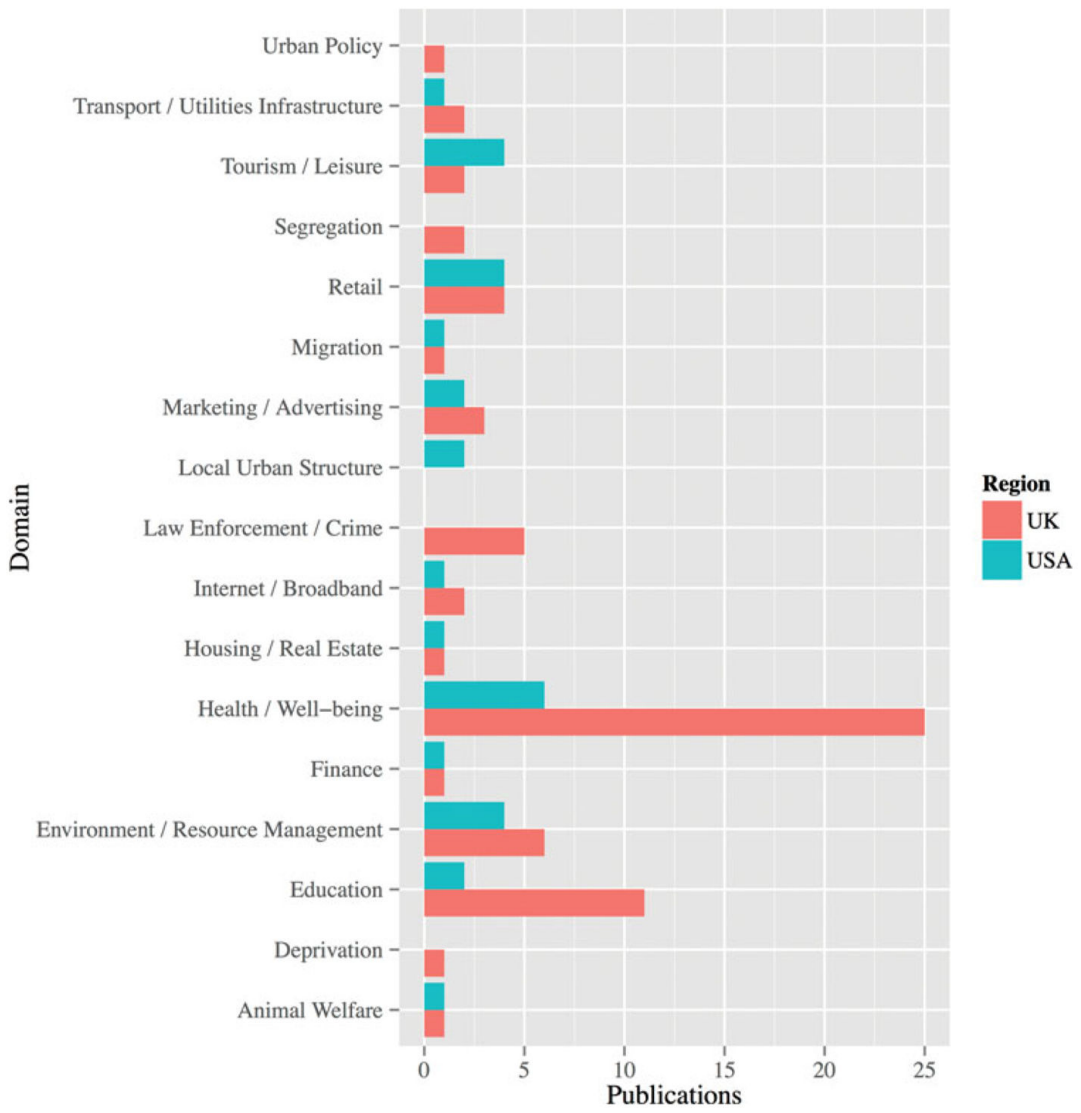
Academic applications of geodemographic systems in the United Kingdom and United States. (Color figure available online.)

The previous section considered the diversity of the U.S. and UK general-purpose classification market, which is now extended to summarize the range and extent of publications that apply geodemographic classifications within the United States and United Kingdom. The results are summarized in Figure 2 and were assembled from extensive online searches of Scopus and Google Scholar (twenty pages of records) for the terms *geodemographics* and *geodemography* and additionally the names of classifications within the United States and United Kingdom. There will undoubtedly be references missing from those extracted; however, we have aimed to be as comprehensive as possible, representing a snapshot as of June 2012. Cross-national comparisons of the academic literature are more complicated than a comparison of the commercial landscape. In the United States, the term geodemographics is less widely used by academics and thus caution should be taken when interpreting the results. Although it is clear that this term is used much more widely in the United Kingdom (sixty-eight articles) than in the United States (thirty articles), these results might not reflect the use of geodemographic methods. The results emphasize that general-purpose geodemographic classifications are more widely used within the United Kingdom. However, in an attempt to capture American literature that has exploited geodemographic methods, but is not explicitly labeled as such, we conducted further searches using the various permutations of the following terms: *neighborhood classification, k-means, census*, and *typology.* These broader search terms uncovered eleven additional articles. The between-country differences do, however, require semantic distinction, chiefly between geodemographic methods and geodemographic systems. A geodemographic system is considered here as comprising a general-purpose classification designed by one party for multiple users and uses, whereas a geodemographic method would focus on the creation of bespoke multivariate typology of geolocated individuals or administrative units for a specific case study area. It should also be noted, however, that there is often overlap between the application of methods and the use of a system and, as such, they cannot be considered to be entirely mutually exclusive.

The themes highlighted in Figure 2 are those that emerged from the searches but include only classifications that might be reasonably called bespoke or applications of geodemographic systems. The use of data reduction methods is widespread in the social sciences, and without a distinction between systems and methods, the comparison becomes untenable and unfocused; for example, the search term *census classification* returned 646,000 records in Google Scholar (accessed 20 December 2012).

This comparative literature search illustrates greater publication activity in the United Kingdom over the United States, but the reasons for this disparity are not entirely clear. Reibel (2011) speculated that both the decline of the factor ecologic approach and the uptake of geodemographic systems in the commercial sector might have inhibited the use of geodemographic methods in academia. Reibel also noted that there is a tendency to favor indexes that measure single variables (segregation, poverty) over discrete multivariate typologies, an observation also corroborated by Abbott (1997) in discussion of the decline of contextual approaches in the social sciences. Another hypothesis is that differences in the use of geodemographic systems in the United States and United Kingdom might relate to variable availability of free geodemographics, either as academically produced models or by commercial organizations offering no-cost access for academic purposes. A lower uptake of geodemographics in academic publications in the United States cannot necessarily be directly linked with classification availability, though, and might simply reflect a lack of demand by academics as opposed to a lack of supply. Within the United Kingdom, over the past ten years there have been reasonable levels of engagement by the commercial geodemographic companies with the academic sector. For example, a number of geodemographic providers made their classifications available to the academic sector without cost, have been directly involved in academic research projects, have had staff with additional honorary positions within educational institutions, or have developed new tailored classifications that overlap into some of the areas in which noncommercial applications commonly occur (e.g., health and education). These types of activities will have encouraged UK academic engagement, but they are not evidenced as strongly in the United States.

A second striking result is that there is such brevity to the academic application areas, with health appearing most prevalently, which could also in part be a function of the number of researchers within this field, and the types of analyses typically conducted. Furthermore, it was identified that geodemographics were applied in a number of different ways, with no particular U.S. or UK bias. These included use for survey sample design, as descriptive measures, as a surrogate for socioeconomic status, as control variables within regression analysis, as a set of levels within multilevel models, as both origin constrains and calibration criteria within spatial interaction models, and, finally, as a measure of small area deprivation.

## Geodemographics as Urban Social Theory?

Geodemographic models can be considered idiographic, providing descriptive characterization of multiple geographical areas; with their operationalization based on the principle that socio-spatial structure is highly correlated with behaviors, attitudes, and preferences. In this way, geodemographic classifications are “theory-free,” as they do not hypothesize a priori about the role of large-scale social mechanisms or individual-level theoretical constructs.

Contemporary research, however, has linked geodemographic classifications to the “spatialization of class” (Parker, Uprichard, and Burrows 2007; Burrows 2008). Sociologists and other social scientists in the United Kingdom have widely utilized classifications based on occupation (e.g., the National Statistics Social Economic Classification) to code individuals into “class”-based groupings. The majority of commercial geodemographic classifications are optimized on the basis of discriminating patterns of consumption (Webber 2007), which have also been shown to have similar stratification by occupational group (Sivadas 1997); as such, it is perhaps not surprising that parallels between these two classification approaches have been drawn. Although non-commercial geodemographics are not explicitly rooted in consumption, implicitly they suggest that meaningful divisions of society can be described through such classification. In the commercial sector, systems are validated through analysis of individual and group-level consumption patterns; for example, Nielsen’s PRIZM system includes hundreds of thousands of individual-level records. Fundamental questions about the relationship among class, status, and consumption are raised by Holt (1998, 1), who suggested that “consumption patterns are no longer consequential to class reproduction,” as these are the outcome of broader social and cultural processes, thus questioning the logic of commercial geodemographic systems. The grand challenge for geodemographic systems is substantiating that they reflect real divisions in society, not chance grouping in the data.

During the mid-1990s, a general body of critique examined the social implications of geographical information systems (GIS; Pickles 1995; Curry 1998) and included the application of geodemographics. Within the context of this critique, Goss (1995) discussed that geodemographic information systems have an instrumental rationality where past differences (as expressed by classifications) can be reproduced through reinforcement of particular behaviors that exist within particular places, an automatic production of space (Thrift and French 2002). Goss sees geodemographic classifications as an oversimplified representation of society that is both defined and sold by marketers, noting that through a “monopoly of market information and control over the means of production of social identity [which is conflated with place in this context] can perhaps engineer the regularity they so desire” (Goss 1995, 162). Goss’s critique of geodemographic systems presumes that consumption plays a critical role in defining identity and social hierarchies. As discussed earlier, however, Holt (1998) questioned this assumption.

Non-commercial systems’ lack of “theory” raises some complications for validation, as without a theory of socio-spatial stratification it remains unclear what attributes these systems should be measured against. Is a good classification one that accurately describes real divisions in society or one that fits a large demographic data set? Are there differences between these two criteria? The most interesting and nuanced considerations of geodemographics grapple with these theoretical questions, for example, by drawing analogies between geodemographics and Bourdieu’s concepts of habitus and field (Tapp and Warren 2010). Webber (2007) also examined the correspondence of a geodemographic classification and the Hall, Marshall, and Lowe (2001) hierarchy of urban centers, demonstrating that at certain levels of hierarchy, there is a tendency for some clusters to be found in greater propensity. These patterns were linked back to sociological processes of gentrification (Lees 2000) and the development of metropolitan habitus (Butler and Robson 2003).

It is clear that there is no grand unifying theoretical framework for geodemographics, and this might pose some problems for validation. However, theory does not have to be seen as exogenous to the classification process, though. Abbott (1997) described the postwar trend in U.S. social sciences as the gradual dominance of the “variables paradigm” over the “contextual paradigm.” Even within the large literature on neighborhood effects on health, crime, education, and well-being, neighborhoods are treated as “bundles of variables” (Galster 2001), rather than discrete classes or types. The focus on variables has contributed what Rybczyński (1996) sees as a poor vocabulary for describing human settlements (contexts), where narrative about urban communities contains a limited number of dimensions, being classified by some combination of urban or suburban, rich or poor, creative, global, measures of capital, or racial segregation. The focus on individual variables as opposed to contexts limits our vocabulary. The value of the contextual approach, as embodied in modern geodemographic classification, is the ability to provide a nuanced picture of large areas, to draw analogy between geographically disparate small areas, and to enable the targeted provision of services (public or commercial). These goals might seem pedestrian in the face of grand social theory, but the geodemographic enterprise holds some theoretical potential. First, one could examine classifications through the lens of theories of social stratification; any correspondence (or lack of correspondence) would have interesting implications for both the theory and the classification in question. Geodemographics as multidimensional descriptors of context also have the potential to provide nuance to public debates about cities, strengthening comparative discourse. If theory is to be embedded within geodemographic classification more significantly, then this will likely need to be led through academic applications, as past methodological practices typically structure current commercial geodemographic systems (Webber 1980).

## Evolving Geodemographics

Going forward, geodemographic classifications face substantial challenges, with institutional shifts in both the United States and United Kingdom changing the nature and availability of the data on which these systems have historically relied. A key input to almost all geodemographic classifications remains the decennial census of the population. This data set has the most extensive coverage of all input data and is collected as a marker of population growth and characteristic change. In both countries, though, these data are not necessary guaranteed to be collected into the future given the growing costs associated with their collection, and additionally, the increasing volume of transactional data that are collected for other purposes, which arguably could provide an effective surrogate (Webber 2009).

In the United Kingdom, the Office for National Statistics (ONS) has launched the “Beyond 2011” consultation that aims to assess alternative options for producing demographic data that are currently demanded by end users of the census in England and Wales. The underlying motivation for this review is a concern over the rising costs of delivery aligned with tighter fiscal constraints and, additionally, the issue of whether the decennial interval is mismatched to demand for more detailed and frequent statistics. The first consultation concluded in January 2012 and was designed to gather end user views on the range of possible alternatives. These results will feed into a second consultation that is taking place in 2013, with final recommendations made during 2014. As such, it is not yet clear what the exact format of the next UK Census will include; however, the ONS has made strong indications that this will be different from a traditional census. Integral to this review will be consideration of the role and availability of administrative data released by the UK government under “open data” licenses. Of particular importance to the future of the UK Census will be the extent to which any disparate open data might be integrated through data linkage within a national information framework (APPSI 2012). A consultation for the provision of such a framework was recently conducted in Scotland (see http://www.scotland.gov.uk/Publications/2012/03/3260). There has been significant interest from the central government in the capitalization of current and future open data resources. For example, in 2012, through the Technology Strategy Board (the UK government’s innovation agency), £10 million of financial backing was provided to establish the Open Data Institute (http://www.theodi.org) with the aim of stimulating new innovations using open data. The extent to which open data will supplement future censuses in the United Kingdom is not yet known, but such substantial government support within an era of austerity should indicate that these developments are being considered seriously.

In the mid-1990s a decision was taken in the United States to split the “long” and “short” form of the U.S. decennial census into separate surveys. The short form of the U.S. Census is constitutionally mandated and is used to apportion each state’s membership in the House of Representatives proportional to their population. The long form asked questions not strictly related to apportionment, including topics such as housing, income, education, commuting, and other variables of considerable value for general policymaking and social science. After more than a decade of testing the long form of the decennial census, in 2010, this was subsumed into a new survey called the American Community Survey (ACS). Whereas the decennial census is constitutionally mandated, the ACS is not and, as a result, it is under constant threat of losing funding. Indeed, during the summer of 2012 the U.S. House of Representatives voted to remove all appropriations for the ACS on the basis that it was an invasion of privacy and an infringement on individual liberty. Although the summer 2012 vote appears to have been largely symbolic, similar debates have occurred in Canada (Shearmur 2010), suggesting a loss of support for public surveys in North America.

In the context of the United States and United Kingdom, these developments will drive a series of challenges for the geodemographics industry. First, in the future, it might not be possible to rely on a base set of data covering demographic characteristics with high spatial resolution. In a U.S. context, the ACS provides estimates down to the block-group level; however, these estimates are so inefficient that they are virtually unusable at this scale (due to wide margins of error). In general, the ACS trades data quality for data frequency, providing annual estimates (based on a five-year moving average) for most U.S. census tracts and block groups. As such, this constant stream of uncertain demographic data raises some novel challenges for geodemographics in the future, and to our knowledge no classification methods account for uncertainty (standard errors) within their input data. In the United Kingdom, basic small-area population estimate data are provided as experimental statistics from the ONS, but they are of lower spatial resolution than those available from the census. Other UK national surveys exist that have potential in this area; however, as yet, these are not routinely used for small area estimation, and indeed it is doubtful that current sample sizes would create usable estimates at higher resolution geographies. For academic classifications using only census data, this could result in them having to be created at much coarser resolutions, limiting potential for geographical comparison. In both the United States and the United Kingdom, it is likely that the commercial sector will be less sensitive to these changes given that they have a lengthy history of compiling large consumer dynamics databases. These databases are populated using a variety of private or commercially sourced data such as credit checking histories, product registrations, private surveys, and public domain data. Although it is impossible to validate the accuracy and completeness of such data given their commercial sensitivity, it is also important to note that these sorts of data linkages have also not been implemented in any uniform way within the public sector in either country. There are, however, isolated cases where linked data from multiple administrative sources have enabled unique insights to be generated; for example, in the area of access to university (Chowdry et al. 2008; Singleton 2010) and in exploring the background of rioters (Home Office 2011). As we enter a post-traditional census era, linkage procedures are likely to become increasingly important to maintain adequate levels of intelligence about the composition, characteristics, and behaviors of local populations; however, in both the United States and United Kingdom, numerous legislative and technical hurdles will need to be overcome for these processes to be effectively implemented.

## Conclusions

In this article we have drawn together an extensive literature on the U.S. and UK origins of geodemographics, outlining how the technique emerged as an extension of earlier attempts to understand urban population structure through social area analysis. Although the origins and development of geodemographics in the United States and United Kingdom have evolved in close parallel, a key difference between the regions is that there has been a reduced prevalence of free or academically produced models in the United States.

An evaluation of the structure of contemporary general-purpose geodemographic classifications revealed that there is a similar level of classification diversity between the United Kingdom and the United States; however, more classifications were supplied in the United Kingdom by small- to medium-sized enterprises. Typologies within the United Kingdom often included three hierarchical levels, whereas in the United States, all classifications studied were limited with two, although in both regions, some classifications were also available with a further and higher level of disaggregation designed to be used when creating bespoke classifications. Finally, the scale of the public domain data typically used to build U.S. geodemographics was much coarser than the UK; additionally, the U.S. classifications had on average greater numbers of clusters at their finest level of disaggregation.

Overall the United Kingdom was shown to have more published academic applications of geodemographics than the United States, and on examining the areas from which these were drawn, it was found that the most prevalent domain of use was health; however, in both regions, applications spanned a wealth of different areas. When exploring how geodemographics were being used within these application areas there was a diversity of analytical procedures ranging from exploratory and descriptive uses through to integration in more complex explanatory models.

Like social area analysis and factorial ecology preceding it, geodemographics were also initiated with a lack of theory underpinning the empirical models. A fundamental theoretical question for geodemographic classification is therefore whether and to what extent differences in observed demographic characteristics align with meaningful “real” divisions in society. Validation is a continuing problem for nonacademic models, but we argue that geodemographics have utility and provide useful descriptors of context, even if they lack a firm theoretical grounding. It is entirely possible that more careful construction and broader use of geodemographic classification in the academy could support the development of a more robust theory of socio-spatial structure.

In the future, the creation of geodemographics in both the United States and United Kingdom is likely to be more complex as the scale and extent of large surveys such as the census comes under increasing fiscal constraint. This is both a challenge and an opportunity for the public and academic sector. In particular, as individual variables become less precise, multidimensional contextual descriptors might be an effective surrogate for detailed variable-by-variable descriptions. In addition, the assembly of rich linked transactional data from the public and private sectors might be used to improve the discriminatory power of geodemographic systems, in spite of imprecise small-area data from public surveys. Similar systems are already in operation within the private sector in the form of consumer dynamics databases, and we argue that it would be very pertinent for knowledge exchange to occur between the sectors.

## Figures and Tables

**Figure 1:**
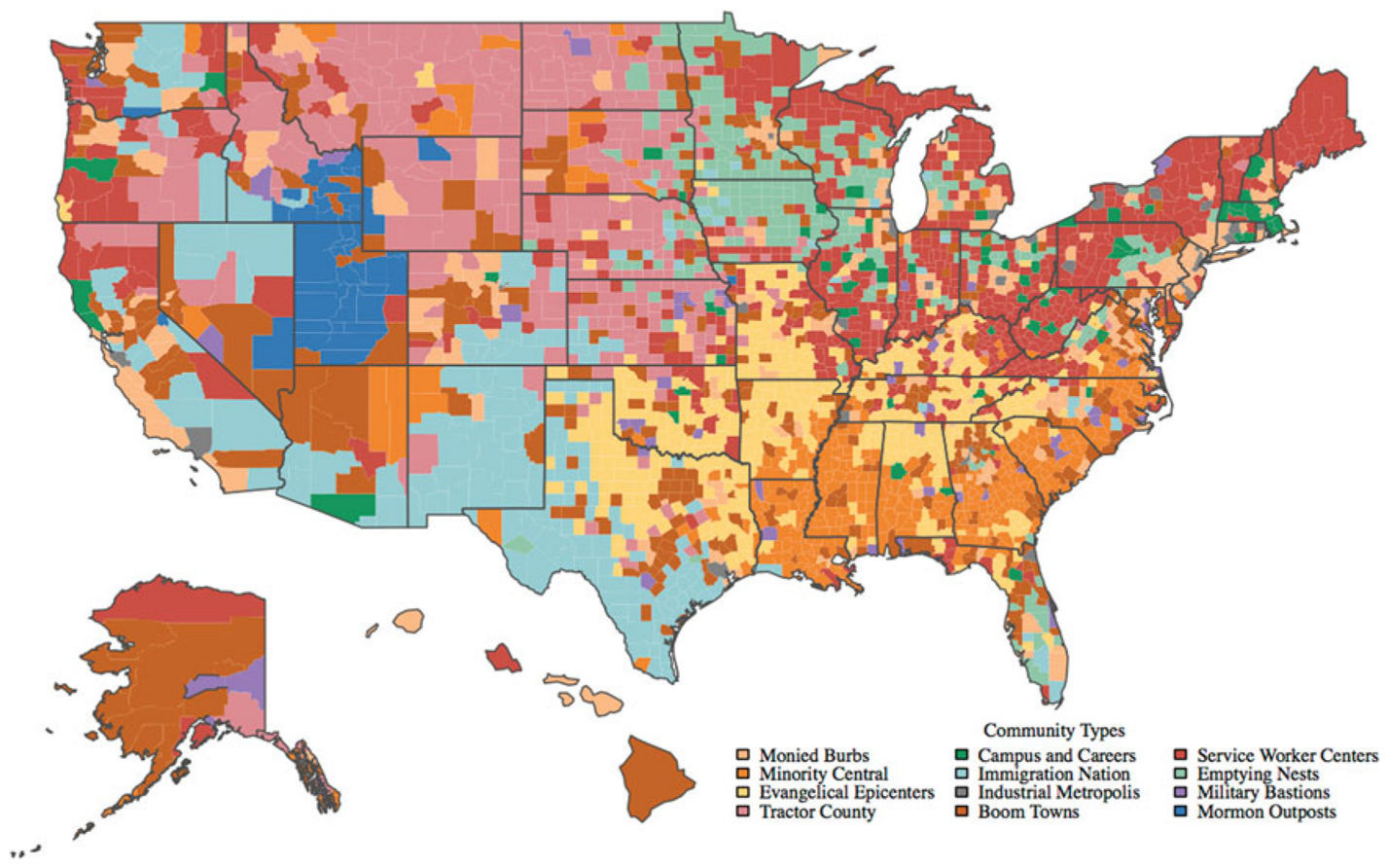
A map showing “Patchwork Nation,” which is an example of a geodemographic classification for the United States. http://www.patchworknation.org (Color figure available online.)

**Table 1:** U.S. general-purpose area-based geodemographic classifications available in 2012

Name	Level 1	Level 2	Micro level	Variables	Taxonomic units	Commercial	SME
Mosaic USA, Experian	19	71		~3,001	ZIP + 4, block group, and ZIP code	Y	N
Nielsen PRIZM	142 or 113	66		Hundreds	Block group, ZIP +4, and larger units	Y	N
Tapestry, ESRI	124 or 115	65		~60	Block group and larger units	Y	N
PSYTE Advantage, Pitney Bowes		72	400		Block group	Y	N
STI Landscape, Synergos Technologies	15	72			Block group	Y	Y
Cohorts, IXI Corporation		30				Y	N
Cameo, Callcredit Information Group	9	52			Block group	Y	N
Acxiom, Personicx	21	70				Y	N
Patchwork Nation, Jefferson Institute	12			125	County	N	N/A
American Clusters, Worldclusters	7	18			Block group	N	N/A

*Note:* SME = small to medium-sized enterprise.

**Table 2:** UK general-purpose area-based geodemographic classifications available in 2012

Name	Level 1	Level 2	Level 3	Micro level	Variables	Taxonomic units	Commercial	SME
Output area classification, Office for National Statistics	7	21	52		41	Output areas	N	N/A
P^2^ People and Places, Beacon Dodsworth	14	41		157	~ 80	Output areas	Y	Y
Mosaic, Experian	15	67		252	440	Unit postcode	Y	N
Acorn, CACI	5	17	56		~ 400	Unit postcode	Y	N
Cameo, Callcredit Information Group	10	58				Unit postcode	Y	N
Cloud Client, Cloud Client Ltd.	15				29	Output area	Y	Y
Sonar, Redmoran	6	24	80		225		Y	Y
Censation, Maw Data Solutions	5	19	53		600	Output area	Y	Y
Personicx Geo, Acxiom	60				~ 400	Postcode	Y	N
Citizen, Marketing Metrix	6	28				Postcode	Y	Y

*Note:* SME = small to medium-sized enterprise.
